# The NIMH intramural healthy volunteer dataset: A comprehensive MEG, MRI, and behavioral resource

**DOI:** 10.1038/s41597-022-01623-9

**Published:** 2022-08-25

**Authors:** Allison C. Nugent, Adam G. Thomas, Margaret Mahoney, Alison Gibbons, Jarrod T. Smith, Antoinette J. Charles, Jacob S. Shaw, Jeffrey D. Stout, Anna M. Namyst, Arshitha Basavaraj, Eric Earl, Travis Riddle, Joseph Snow, Shruti Japee, Adriana J. Pavletic, Stephen Sinclair, Vinai Roopchansingh, Peter A. Bandettini, Joyce Chung

**Affiliations:** 1 Magnetoencephalography Core Facility, National Institute of Mental Health, NIH, DHHS, Bethesda, MD USA; 2 Data Science and Sharing Team, National Institute of Mental Health, NIH, DHHS, Bethesda, MD USA; 3 Office of the Clinical Director, National Institute of Mental Health, NIH, DHHS, Bethesda, MD USA; 4 Laboratory of Brain and Cognition, National Institute of Mental Health, NIH, DHHS, Bethesda, MD USA; 5 Functional MRI Facility, National Institute of Mental Health, NIH, DHHS, Bethesda, MD USA; 6 Section on Functional Imaging Methods, National Institute of Mental Health, NIH, DHHS, Bethesda, MD USA

**Keywords:** Cognitive neuroscience, Biomarkers, Databases

## Abstract

The NIMH Healthy Research Volunteer Dataset is a collection of phenotypic data characterizing healthy research volunteers using clinical assessments such as assays of blood and urine, mental health assessments, diagnostic and dimensional measures of mental health, cognitive and neuropsychological functioning, structural and functional magnetic resonance imaging (MRI), along with diffusion tensor imaging (DTI), and a comprehensive magnetoencephalography battery (MEG). In addition, blood samples of healthy volunteers are banked for future analyses. All data collected in this protocol are broadly shared in the OpenNeuro repository, in the Brain Imaging Data Structure (BIDS) format. In addition, task paradigms and basic pre-processing scripts are shared on GitHub. There are currently few open access MEG datasets, and multimodal neuroimaging datasets are even more rare. Due to its depth of characterization of a healthy population in terms of brain health, this dataset may contribute to a wide array of secondary investigations of non-clinical and clinical research questions.

## Abstract (summary)

**Table:** 

Measurement(s)	brain activity • Blood Oxygen Level-Dependent Functional MRI • regional part of brain • cerebral white matter structure • cerebral blood flow
Technology Type(s)	Magnetoencephalography • Functional Magnetic Resonance Imaging • Magnetic Resonance Imaging • Diffusion Tensor Imaging • Arterial Spin Labeling Magnetic Resonance Imaging
Sample Characteristic - Organism	Homo sapiens
Sample Characteristic - Location	United States of America

## Background & Summary

The disciplines of cognitive and affective neuroscience, as well as psychiatry, investigate the neurobiological mechanisms underlying human cognition and emotion. Broad yet deep datasets containing indices of brain and neural functioning, cognition, and emotional functioning in healthy volunteers are a vital resource. These datasets can be used to investigate questions such as which brain circuits underlie basic cognitive processes, how brain activity is correlated with behavior across the spectrum of healthy brain function, and how macroscale brain organization relates to function. Importantly, multimodal data collected on healthy volunteers can also be interrogated to address questions about abnormal human cognitive and emotional functioning found in psychiatric disorders. Multifaceted and overlapping cognitive manifestations are observed in disorders as diverse as major depressive disorder^1,2^, schizophrenia^3^, autism spectrum disorders^4^, attention deficit hyperactivity disorder (ADHD)^5,6^, and substance disorders^6,7^ although it is unclear if cognitive abnormalities precede development of a disorder, or if cognitive deficits are a consequence of the disorder.

Although there are numerous shared neuroimaging data repositories, few incorporate the breadth of modalities and the inclusion of a wide range of clinical information on healthy research volunteers. Of existing datasets including both MRI and MEG components, several merit mention. The Human Connectome Project (HCP)^8^, the Cambridge Centre for Ageing and Neuroscience Cam-CAN dataset^9^, and the Mother of Unification Studies (MOUS) repositories all contain structural and functional MRI (fMRI) and MEG data^10^. The Open MEG Archive (OMEGA)^11^ and MEG-UK project (not yet released) contain MEG and structural MRI datasets. While additional repositories with both MEG and MRI data exist, such as the Federal Interagency Traumatic Brain Injury Research Informatics System (FITBIR; https://fitbir.nih.gov) and NIMH Data Archive (NDA; https://nda.nih.gov), the data typically come from multiple small studies with little standardization of acquisition methods or data format. Other large scale datasets merit mention, such as the Lifebrain project (https://lifebrain.uio.no), integrating 11 European brain imaging cohorts, and the UK Biobank (https://www.ukbiobank.ac.uk). Importantly, these datasets incorporate longitudinal measurements which are of obvious importance to the field.

The dataset collected and shared under the NIMH Healthy Research Volunteer (RV) Study (Recruitment and Characterization of Healthy Research Volunteer for NIMH Intramural Studies NCT033046) focuses on characterizing a healthy volunteer cohort for studies investigating the neurobiology of psychiatric illness. The assessments for the study prioritize brain health but also screen for clinical conditions that may affect biological studies of mental health such as prior brain injury, drug use, or psychotropic medications. While the focus on healthy volunteers may limit generalizability, our hope is that we oversample characteristics specifically related to brain health in relation to neuropsychiatric disease. The NIMH RV dataset is also distinguished by the extent of information collected about each participant. All collected data are anonymized/de-identified (to the greatest extent possible while maintaining utility) and shared with the research community. The shared data include online self-report survey data used for initial screening of health, and in-person clinical assessments of medical and mental health, laboratory tests of blood and urine, IQ assessment, family history, and surveys of anxiety and depressive symptoms on those who are found to be eligible. A subset of healthy volunteers receive structural MRI, diffusion tensor imaging (DTI), resting state fMRI, and MEG. The breadth of the MEG task battery, with its coverage of multiple cognitive domains, makes the dataset especially valuable. Importantly, data collection is ongoing; while data from 1,090 participants (284 with in-person assessments, 155 with MRI, and 67 with MEG) are currently shared, updated data from newly enrolled participants will be added regularly. The purpose of this data descriptor is to introduce and comprehensively describe the broadly shared NIMH RV dataset, to facilitate research in the greater scientific community, particularly those conducting neuroimaging studies.

## Methods

### Recruitment and online screening

This study is a convenience sample of healthy persons in the DC metropolitan area interested in participating in research. Inclusion criteria for the study require that participants are adults at or over 18 years of age in good health with the ability to read, speak, understand, and provide consent in English. All participants provided electronic informed consent for online screening and written informed consent for all other procedures. Exclusion criteria include a history of significant or unstable medical or mental health condition requiring treatment; current self-injury, suicidal thoughts or behavior; current illicit drug use by history or urine drug screen; abnormal physical exam or laboratory result at the time of in-person assessment; or less than an 8^th^ grade education or IQ below 70. Current employees, or first-degree relatives of NIMH employees are excluded, although other NIH employees may participate. Study participants are recruited through direct mailings, bulletin boards and listservs, outreach exhibits, print advertisements, and electronic media. Supplemental Table 1 gives a comparison of demographics for the current dataset with demographics from Montgomery County, MD, the location of NIMH. The NIMH sample was somewhat younger and more female than the surrounding population.

All potential volunteers first visit the study website (https://nimhresearchvolunteer.ctss.nih.gov), check a box indicating consent, and complete preliminary self-report screening questionnaires. The study website is HIPAA compliant and therefore does not collect personally identifiable information (PII); instead, participants are instructed to contact the study team to provide their identity and contact information. The questionnaires include demographics, clinical history including medications, disability status (WHODAS 2.0), mental health symptoms (modified DSM-5 Self-Rated Level 1 Cross-Cutting Symptom Measure), substance use survey (DSM-5 Level 2), alcohol use (AUDIT), handedness (Edinburgh Handedness Inventory), and perceived health ratings. At the conclusion of the questionnaires, participants are again prompted to send an email to the study team. Survey results, supplemented by NIH medical records review (if present), are reviewed by the study team, who determines if the participant is likely eligible to participate as a healthy volunteer based on the inclusion/exclusion criteria. These participants are then scheduled for an in-person assessment. Follow-up phone screenings are also used to determine if participants are eligible for in-person screening.

### In-person assessments

At this visit, participants undergo a comprehensive clinical evaluation to determine final eligibility to be included as a healthy research volunteer. The mental health evaluation consists of a psychiatric diagnostic interview (Structured Clinical Interview for DSM-5 Disorders (SCID-5), along with self-report surveys of mood (Beck Depression Inventory-II (BD-II) and anxiety (Beck Anxiety Inventory, BAI) symptoms. An intelligence quotient (IQ) estimation is determined with the Kaufman Brief Intelligence Test, Second Edition (KBIT-2) (n = 267). The KBIT-2 is a brief (20–30 minute) assessment of intellectual functioning administered by a trained examiner. There are three subtests, including verbal knowledge, riddles, and matrices.

Medical evaluation includes medical history elicitation and systematic review of systems. Biological and physiological measures include vital signs (temperature, blood pressure, pulse), as well as weight, height, and BMI. Blood and urine samples are taken and a complete blood count, acute care panel, hepatic panel, thyroid stimulating hormone, viral markers (HCV, HBV, HIV), C-reactive protein, creatine kinase, urine drug screen, and urine pregnancy tests are performed. In addition, blood samples that can be used for future genomic analysis, development of lymphoblastic cell lines, or other biomarker measures are collected and banked with the NIMH Repository and Genomics Resource (Infinity BiologiX). Any future assessments on stored samples will be shared as they are available. The Family Interview for Genetic Studies (FIGS) was later added to the assessment in order to provide better pedigree information; the Adverse Childhood Events (ACEs) survey was also added to better characterize potential risk factors for psychopathology. The entirety of the in-person assessment not only collects information relevant for eligibility determination, but it also provides a comprehensive set of standardized clinical measures of volunteer health that can be used for secondary research.

### MRI Scan

Participants who were determined to be eligible for inclusion as healthy research volunteers based on the in-person assessment are given the option to consent for a magnetic resonance imaging (MRI) scan, which can serve as a baseline clinical scan to determine normative brain structure as well as a research scan with the addition of functional sequences (resting state and diffusion tensor imaging). Details of scan types are given below and in Table 2.The MR protocol used was initially based on the ADNI-3 basic protocol^12^, but was later modified to include portions of the ABCD^13^ protocol. Because there may be small changes in parameters from the standard ABCD/ADNI3 sequences, detailed sequence descriptions are shared in the BIDS sourcedata directory. Additional images collected with parameters inconsistent with the primary dataset are also shared in the sourcedata directory with detailed metadata files so that investigators can include them in analyses at their discretion; numbers for each scan type are given in Supplemental Table 2. High resolution hippocampal scans were originally part of the battery but removed due to time constraints, thus all collected scans are in sourcedata. Some images were acquired with and without GE’s proprietary surface coil intensity correction algorithm applied, these are designated “rec-SCIC” in the repository. Wherever available, both image types were shared. Scan types are as follows, please refer to Table 3 for numbers:

- The T1 scan from ADNI3 (fSPGR) was initially acquired, but was later replaced the T1 scan from ABCD (MPRAGE)
- The 2D FLAIR sequence from ADNI2
- The 3D FLAIR sequence from ADNI3, altered to match the resolution and geometry of the T1 scan (this scan was optional)
- The ADNI3 T2* weighted scan
- The 3D T2 weighted scan from the ABCD protocol resolution and bandwidth matched to the T1 scan
- The ADNI3 pCASL scan, altered to add fat saturation
- The DTI scan from ADNI3 was modified to include the slice-select gradient reversal method (for 24 directions) and to turn reconstruction interpolation off.
- The eyes-open resting state from ADNI3 was modified to use a TE of 16.9 ms and was acquired together with 1- phase-encoding reversed volumes
- Field maps for both DTI and rsfMRI were acquired

On the same visit as the MRI scan, volunteers are administered a subset of tasks from the NIH Toolbox Cognition Battery. The four tasks asses attention and executive functioning (Flanker Inhibitory Control and Attention Task), executive functioning (Dimensional Change Card Sort Task), episodic memory (Picture Sequence Memory Task), and working memory (List Sorting Working Memory Task).

### MEG recording

An optional MEG study was added to the protocol approximately one year after the study was initiated, thus there are relatively fewer MEG recordings in comparison to the MRI dataset. All participants eligible for MRI who did not have contraindications such as implanted metal or dental hardware (which would reduce data quality) were offered participation in MEG. MEG studies are performed on a 275 channel CTF MEG system (CTF MEG, Coquiltam BC, Canada), using third-order gradient balancing for noise correction. All datasets were collected at a sampling rate of 1200 Hz, with a quarter-Nyquist filter of 300 Hz. The position of the head is localized at the beginning and end of each recording using three fiducial coils. These coils are placed 1.5 cm above the nasion, and at each ear, 1.5 cm from the tragus on a line between the tragus and the outer canthus of the eye. For 50 participants, photographs were taken of the three coils and used to mark the points on the T1 weighted structural MRI scan for co-registration. For the remainder of the participants (n = 17), a Brainsight neuronavigation system (Rogue Research, Montréal, Québec, Canada) was used to coregister the MRI and fiducial localizer coils in real-time prior to MEG data acquisition.

The MEG task battery was designed to assess multiple cognitive domains which are relevant to neuropsychiatric disorders. The MEG battery is divided into two parts, with additional participant/equipment set-up in between. All tasks are coded in either PsychoPy or Presentation software and are available on GitHub (https://github.com/nih-megcore/hv_protocol). In Supplemental Table 3, we give the number of trials per condition and timing for each task, with a summary of the tasks presented here. Accuracy and reaction time can be calculated from the marker timing files for all tasks. Prior to any of the tasks, a brief “artifact” recording was acquired. Participants were asked to blink, move their eyes, breathe deeply, clench their jaw, and swallow. The first half of the MEG session consists of a modified Hariri Hammer task, a modified Sternberg task, and a resting state acquisition. The order is counterbalanced across subjects, with either the Hariri Hammer or Sternberg occurring first or last, and the resting state acquisition always acquired between the two tasks. The Hariri Hammer task was originally developed for fMRI^14^, and later adapted for MEG^15^ as a sensitive probe of amygdala function. The original task presents three faces in a triangle, with the target stimuli on the top and match stimuli below. The participant is required to select from the two faces on the bottom to match the emotion of the target stimuli above. We further adapted the task by temporally separating the target and match stimuli with a small delay and fixation cross. The target face is presented first alone, centrally, followed by a fixation cross, and then the two (probe) faces presented centrally as a pair. The delay was incorporated to isolate the evoked response to a single emotional face. This change allows studies investigating whether the trial-to-trial variation in the magnitude or duration of the response to the first face affects the reaction time or accuracy of the choice, and whether this relationship is modulated by emotion. The Sternberg task was developed as a probe of working memory^16^, and has been previously used in MEG^17^. The original version sequentially presents a series of digits or letters, and after a delay, presents a single digit or letter, and the participant is asked to indicate whether the letter or digit appeared in the series. The version used here presents the series in its entirety, rather than sequentially, in order to reduce the time required to complete the task. We present two conditions: four-letter strings and six-letter strings. The resting state scan is 6 minutes in duration, and participants are given no specific instructions other than to close their eyes and remain still.

Following a brief break to set up stimulus delivery equipment, participants receive a somatosensory task. A brief tactile stimulus is delivered to the right index finger using pneumatic pressure on a thin flexible membrane. In order to measure both the response to the stimulus as well as the expectation of the stimulus, 15% of all stimuli are omitted. Following the somatosensory task, participants perform either a go/no-go task or a three-stimulus oddball task. Order is counterbalanced across participants, and a naturalistic viewing task is always performed between the two. The go/no-go task was similar to previous implementations for MEG^18^. Briefly, participants are rapidly presented outlines of shapes, and respond to every shape unless there is an “X” in the middle. The three-stimulus oddball consists of three stimuli presented in one of four randomized orders. There is a standard tone, a higher-pitched rare tone, and a white noise stimulus; participants are asked to respond via button press to the high-pitched rare tone. The naturalistic viewing task consists of the approximately 9-minute short film “Growth,” by Sil van der Woerd (https://www.silvanderwoerd.com/growth). The film contains audio, but no dialogue, and is presented in its entirety (excluding the credits, which were distributed on paper to all participants after the session).

In addition to the subject datasets, we additionally acquire empty room datasets. Because the CTF sensors are quite stable, we collect these scans approximately monthly. The scans are 100 s in duration, collected with a sampling frequency of 4800 Hz. Although the BIDS specification recommends that these datasets be placed in a directory separate from research participant data, and organized by date, this is unsatisfactory because we have removed the data of scan from the human datasets. In order to maximize ease of use for researchers, we have placed the appropriate empty room dataset in the same directory as the participant MEG data. Because empty room datasets were not acquired before every scan, some subject directories will share the same empty room dataset.

### Preprocessing methods

We distribute the data in a minimally processed, raw format. However, in order to facilitate data analysis, the MRI data are converted to NIfTI and transformed into BIDS format^19^ using Dcm2Bids version 2.1.6 (https://github.com/UNFmontreal/Dcm2Bids/releases/tag/2.1.6), which is a wrapper for dcm2niix version 1.0.20211006 (https://github.com/rordenlab/dcm2niix)^20^. To preserve subject privacy, structural MRI scans are defaced using AFNI Refacer version 2.31 (https://afni.nimh.nih.gov/pub/dist/doc/htmldoc/tutorials/refacer/refacer_run.html). First with AFNI Refacer, a single T1-weighted image was defaced from each of the 155 participants and a defaced image and mask were produced. Next every other image was rigid-body-aligned to its participant’s defaced image with a mutual information cost function using FSL’s FLIRT^21–23^. Last, the deface mask was aligned using the FLIRT alignment matrix to each subject’s other images. The alignment procedure worked well in all cases except one. That one image was instead masked with no registration as it overlaid quite well on the originally defaced image.

The MEG data were transformed to BIDS format using MNE-BIDS (https://github.com/mne-tools/mne-bids) {Appelhoff, 2019 #36;Niso, 2018 #38} and additional python scripts written locally. For all MEG tasks, we ran custom python scripts that generated marks for all stimulus types for the cognitive tasks (these are available as a git submodule called “hv_proc” in https://github.com/nih-megcore/hv_protocol). Because the data is shared in the proprietary CTF “.ds” data structure format, these markers are saved within the “.ds” directory in a file entitled Marker.mrk. In addition, the markers are also stored in “events.tsv” sidecar files in the meg directory according to the BIDS MEG specification. While the stimulus delivery software sends triggers indicating the type of stimulus, these are not coincident with the appearance on screen due to delays induced by the refresh rate of the projector. A ProPixx projector was used for all visual presentation, and the upper left pixel of the projected image can be used to encode the precise time at which the stimulus is displayed. Thus, all marks we indicate in the recordings have been adjusted so that they coincide with the precise time of display. In addition, marks for somatosensory and auditory stimuli have been adjusted for delays in stimulus delivery by subtracting the mean measured delay between parallel port onset and stimulus delivery.

We distribute the location of the MEG fiducial coils in the coordinate space of the anatomical MRI. No other pre-processing or filtering was performed.

## Data Records

**Fig. 1:**
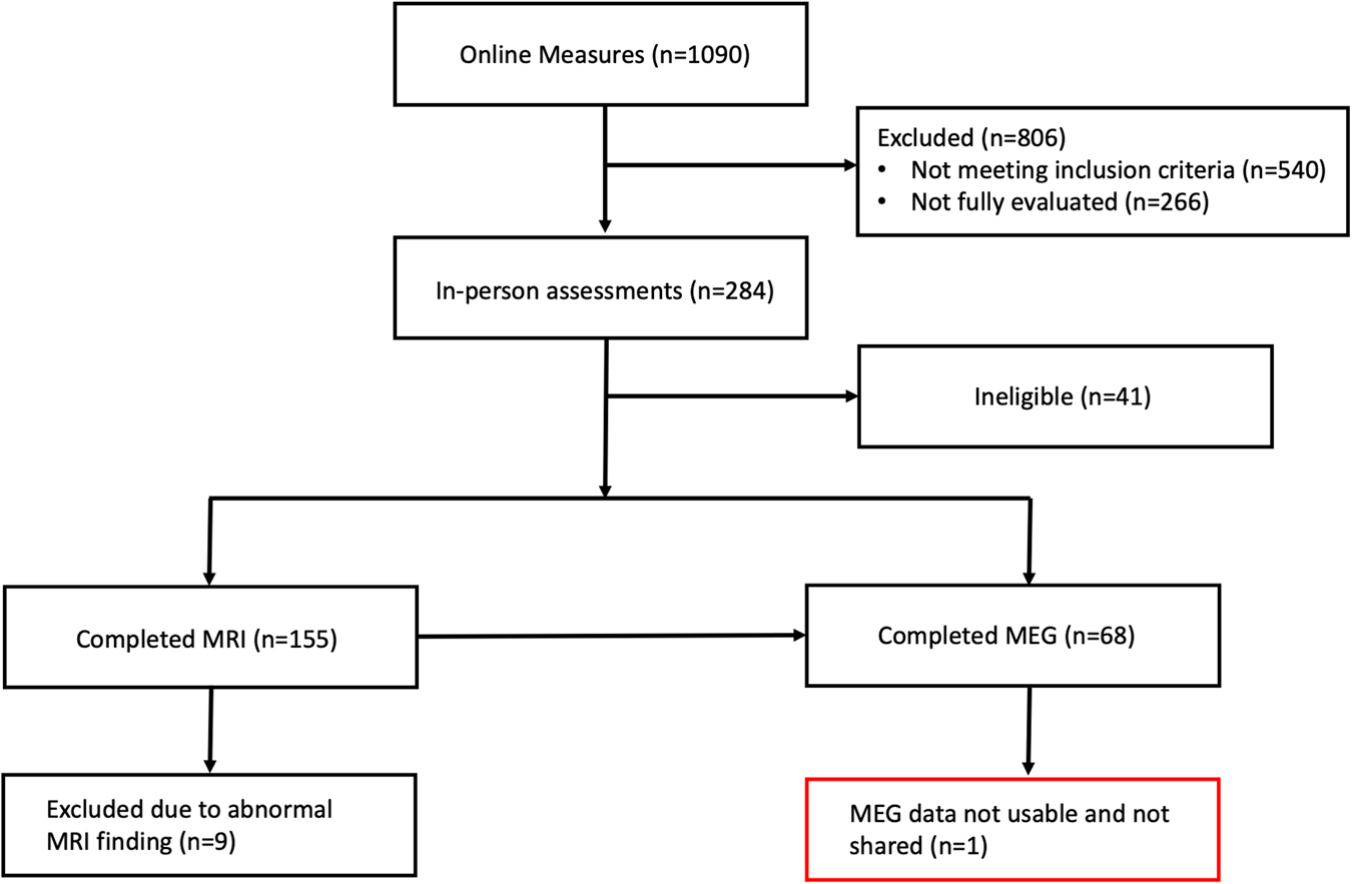
A consort-style diagram illustrating the participant flow through the study. Participants who did not provide unambiguous contact information or a minimal set of demographic responses are not shown. One collected MEG recording is not shared due to extremely poor data quality. The intention was to perform MEG only on participants with MRI scans, but due to scheduling and unanticipated contraindications, two MEG participants do not have MRI scans.

**Fig. 2:**
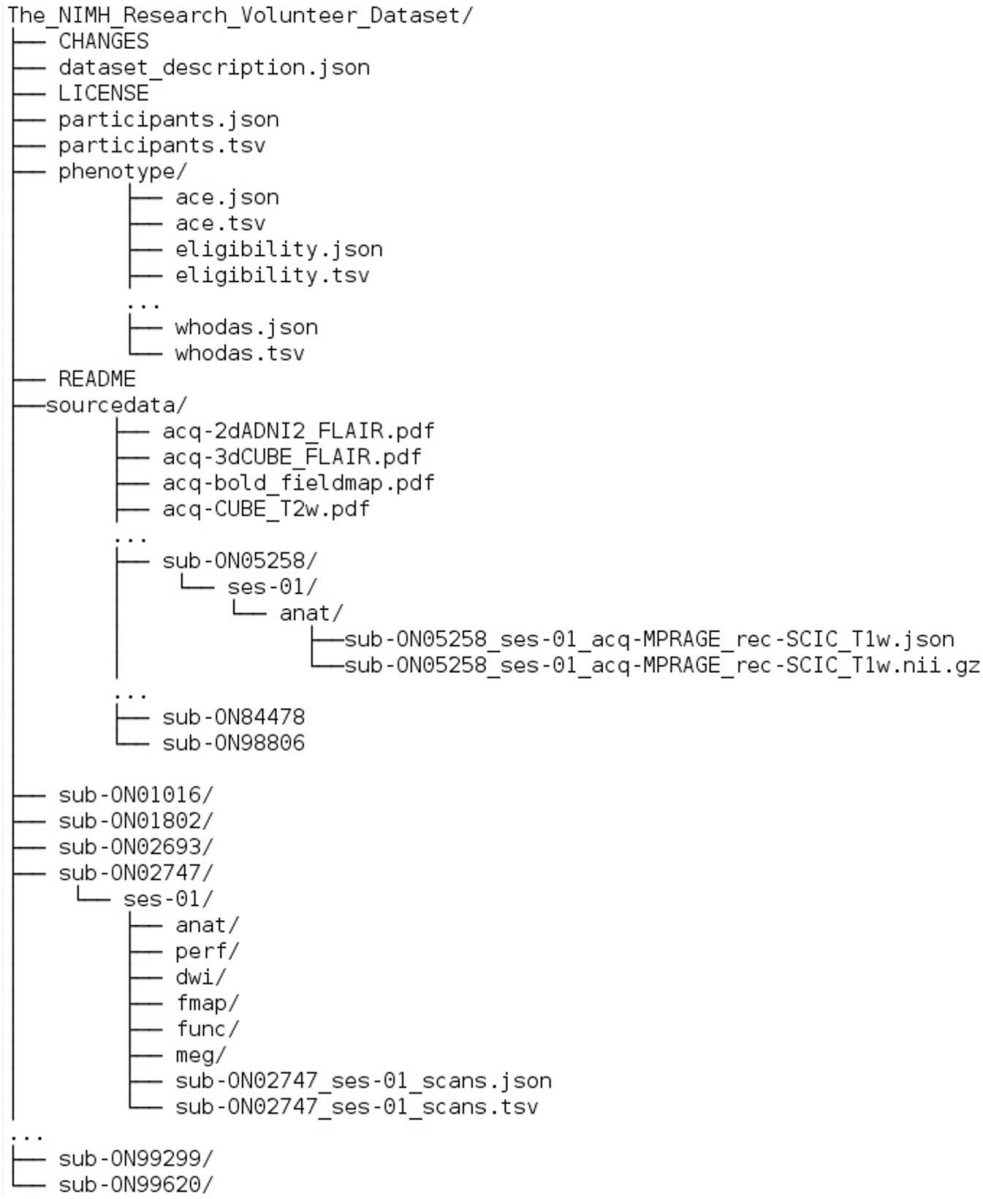
Illustration of the BIDS tree for the dataset. Some files in the phenotype directory are not shown for clarity.

The NIMH RV dataset can be accessed via OpenNeuro (10.18112/openneuro.ds004215.v1.0.0^24^). A consort-style diagram for participant flow is shown in Fig. 1. To minimize including potentially fraudulent or duplicate records, we only count participants who signed the e-consent, provided at least age or gender on the online measures, and provided contact information to the study team. We include participants who signed e-consent from the opening of the protocol to 6/3/2020, at which point it became evident that in-person assessments would likely be suspended for an extended period due to the COVID-19 pandemic. This hiatus was a natural point at which to prepare the first release of data from the study. In-person assessments as of December 2021 and data collection is on-going, with annual releases planned approximately annually. The data on the 1,090 healthy research volunteers from the first release are available on the OpenNeuro platform^24^; additional releases will utilize the same platform. Given the staged release of data, we anticipate that reliability and replicability analyses can be performed using the present release dataset and future releases. An image of the BIDS tree appears in Fig. 2. Demographics for all participants, and the subgroup of participants with MRI or MEG imaging appears in Table 1. Note that the demographics table includes all participants, irrespective of whether or not they were found to be healthy on all screening assessments.

**Table 1:** Demographics for all participants, as well as the subsets of participants with MRI or MEG.

	Total		MRI		MEG	
Sample Size	1,090		155		67	
Age (Mean/Standard Deviation)	38.46	15.47	34.05	12.75	34.16	11.97
Age (Median/Min-Max)	33	18–89	30	18–72	32	20–64
Female (N/%)	707	65.20%	102	65.81%	46	68.66%
Male (N/%)	377	34.59%	53	34.19%	21	31.34%
**Race/Ethnicity**						
American Indian or Alaskan Native (N/%)	5	0.46%	0	0.00%	0	0.00%
Asian (N/%)	110	10.17%	25	16.13%	12	17.91%
Black/African American (N/%)	157	14.53%	22	14.19%	13	19.40%
White (N/%)	751	69.47%	101	65.16%	40	59.70%
Multiple Races (N/%)	52	4.81%	7	4.52%	2	2.99%
Ethnicity: Hispanic/Latino? (N/%)	96	9.02%	10	6.71%	5	7.69%
**Handedness**						
Hand (Right) (N/%)	919	84.31%	134	86.45%	60	89.55%
Hand (Left) (N/%)	64	5.87%	7	4.52%	3	4.48%
Hand (Ambidextrous) (N/%)	84	7.71%	14	9.03%	4	5.97%

Note that age is unknown for 10 participants, sex is unknown for 6 participants, handedness is unknown for 23 participants, race is unknown for 6 participants, and ethnicity is unknown for 8 participants. Participants were allowed to endorse more than one race/ethnicity; the table reflects the primary endorsement.

The number of records for the behavioral and clinical measures from the online surveys and from the in-person evaluations are shown in Table 2. As stated previously, the MRI scanning protocol was updated after data acquisition had begun; thus, the number of participants completing each scan varies. Some scans were transitioned from the ADNI3 battery to the ABCD protocols. Most subjects completed the full 60 minutes battery of scanning. For some participants the scan needed to be terminated early. Typically, this resulted in the resting state scan being dropped; details of the number of datasets appear in Table 3. Table 4 lists the individual components of the MEG sessions. Similar to the MRI dataset, for some participants a task was omitted from the battery due to time constraints or technical issues.

**Table 2:** Online and In-person behavioral and clinical measures, along with the corresponding datafile in the BIDS repository.

	Measure	File Name	N Total	MRI	MEG
**Online**	Demographics	demographics	1090	155	67
	World Health Organization Disability Assessment Schedule 2.0 (WHODAS 2.0)	whodas	1077	155	67
	DSM-5 Self-Rated Level 1 Cross-Cutting Symptoms Measure – Adult (modified)	mental_health_questions	1072	155	67
	DSM-5 Level 2 Substance Use - Adult	drug_use	1069	155	67
	Alcohol Use Disorders Identification Test (AUDIT)	audit	1068	155	67
	Edinburgh Handedness Inventory (EHI)	ehi	1067	155	67
	Health History Form	health_history_questions	1058	155	67
	Perceived Health Rating - self	health_rating	1047	154	67
**In-Person**	Medical and mental health diagnosis, medications, physical exam, lab findings, vital signs, BMI	clinical_variable_form	272	148	61
	Beck Depression Inventory-II (BDI-II)	bdi	273	149	64
	Beck Anxiety Inventory (BAI)	bai	271	148	63
	Kaufman Brief Intelligence Test 2nd Edition (KBIT-2) and Visual Analogue of Effort Scale (VAS)	kbit2_vas	267	152	67
	Perceived Health Rating - clinician	perceived_health_rating	266	145	63
	Creatine Kinase, C-reactive Protein, TSH	blood_chemistry	264	143	58
	Urine drug screen and pregnancy test (if indicated)	urine_chemistry	262	143	60
	Viral markers (Hepatitis B, C and HIV)	other	259	140	55
	Complete Blood Count with Differential	cbc_with_differential	258	139	56
	Chemistry Panel	acute_care	255	138	54
	Hepatic Panel	hepatic	253	136	53
	Structured Clinical Interview for DSM-5 Disorders (SCID-5)	scid5	247	136	60
	Family Interview for Genetic Studies (FIGS)	figs	153	90	53
	NIH Toolbox measures	nih_toolbox	157	144	63
	MRI Variables form	mri_variables	90	81	46
	Adverse Childhood Experiences (ACEs)	ace	49	20	13
	Research participation satisfaction survey	satisfaction	38	24	4

Number of records for each assessment given.

**Table 3:** Name of each MRI scan and the number of participants completing each.

Modality	Filename	Image Notes	Count
T1w	acq-FSPGR_rec-SCIC_T1w	only SCIC available	62
	acq-MPRAGE_rec-SCIC_T1w	SCIC + Orig shared	89
	acq-SagittalMPRAGE_T1w	SCIC + Orig shared	4
2D FLAIR	2dADNI2_rec-SCIC_FLAIR	SCIC + Orig shared	146
3D FLAIR	acq-3dCUBE_rec-SCIC_FLAIR	SCIC + Orig shared	25
T2w CUBE	acq-CUBE_rec-SCIC_T2w	Orig + some SCIC shared	153
T2*	rec-SCIC_T2starw	only SCIC available	147
ASL	asl	eyes open	142
DTI	dir-unflipped_dwi	24 directions (reverse)	134
	dir-flipped_dwi	48 directions (primary)	134
rsfMRI	task-rest_dir-forward_bold	Forward	130
	task-rest_dir-reverse_bold	Reverse	129
DTI Field Map	acq-dwi_fieldmap		66
fMRI Field Map	acq-bold_fieldmap		59

Note that wherever possible, both original and SCIC corrected images are shared.

**Table 4:** Name of each MEG recording and the number of participants completing each.

MEG Task	N
Artifact	67
Hariri Hammer	65
Sternberg	65
Somatosensory	64
Resting State (eyes closed)	67
Go/No-go	64
Naturalistic Viewing	63
Three Stimulus Oddball	63

## Technical Validation

### Participant eligibility

It is our intention to maximize the amount of data available for download. All participants who provided a minimal set of data in the online questionnaire and provided unambiguous contact information to the investigators are shared. Eligibility is given in the file *eligibility.tsv* in the BIDS distribution. Participants are marked as eligible, ineligible, or unknown. Participants that were found to be healthy volunteers based on all assessments (online, in-person, and MRI, if performed) were listed as “eligible.” The unknown category includes participants who the team had difficulty scheduling, participants who were scheduled to be evaluated or responded during the time of the COVID-19 pandemic, and participants who failed to complete all the online measures. For participants ruled ineligible, they are coded as ineligible due to medical, mental health, MRI, or other reasons. Data from all participants who came for an in-person portion of the study are shared. Some of these participants were found ineligible for further participation due to clinical findings on the SCID-5 or medical findings (or declined further participation). Only those participants who were found to be medically and psychiatrically healthy and wished to continue in the study participated in the MRI and MEG portions of the study. As described above, nine participants who had an incidental finding on the MRI scan are shared but marked as ineligible. One participant had unusable MEG data; all data for that participant is shared with the exception of the MEG recording. We recognize that performing imaging only on participants meeting medical and psychiatric eligibility criteria reduces generalizability. While we were somewhat limited by the finite imaging resources available, our intention was also to characterize brain health relative to both medical and psychiatric illness. This necessitated ensuring that all participants met stringent criteria for health. While we recognize that the full spectrum of normal physiology may not be captured, we hope that our inclusion criteria may lead to oversampling of characteristics related to brain health. Future data collection projects using the same paradigms may consider a wider sampling of the healthy population.

### Behavioral data

Due to issues with an early version of the Edinburgh Handedness Inventory, this scale has data from somewhat fewer participants than the other scales administered during screening, although handedness was also assessed by self-report.

### MRI data

Defaced scans were visually inspected for quality control (QC) by two raters using VisualQC version 0.6.1 (https://github.com/raamana/visualqc), first with *vqcdeface* for the 155 defaced images, and then *vqcalign* for the 723 image alignments. All MRI data passed QC and are shared without further pre-processing.

### MEG data

The intention was to perform MEG only on participants with MRI scans, but due to scheduling and unanticipated contraindications, two MEG participants do not have MRI scans. As noted previously, all participants were recorded while being asked to generate artifacts. These recordings can potentially be used to generate automated or semi-automated artifact detection and/or removal algorithms. Head location was collected at the beginning and end of every recording. Continuous head localization was not utilized due to issues with the function of the system and may be added for future recordings. Average motion during the recording is stored in the *coordsystem.json* file and*.hc* file in the BIDS data directory. Total head motion is not calculated, and all recordings are shared, regardless of motion so that individual investigators can decide what threshold is appropriate for their study. Every effort was made to have every participant complete all the tasks; however, due to time constraints tasks occasionally had to be dropped. Thus, the total number of datasets for each task is given in Table 4. For an initial subset of recordings, there were issues in the way stimulus onset triggers were sent to the acquisition software for the Hariri Hammer and the Sternberg tasks. However, the detailed PsychoPy log files enabled the correct markers to be placed in the dataset.

## Usage Notes

This data collection is available at OpenNeuro^24^. Data is broadly shared, under a Creative Commons Zero (CC0) license.

### MRI data

Image filenames for each modality appear in Table 3, along with usage notes. The 48-direction DTI images were labeled automatically by the dcm2niix converter as “unflipped” based upon the PhaseEncodingPolarity parameter, although these should be considered the primary images. The 24-direction image sets can be used as the reverse polarity for analysis.

### MEG data

As noted in the pre-processing section above, we parsed the logfiles produced by the task delivery software to place markers in the dataset. For visual stimuli, we utilized the projector channel to correct the time of each mark so it corresponded to the time at which the stimulus appeared on the participant’s screen rather than the time at which the trigger was sent. We attempted to mark all task relevant features, such that any potential contrast of interest can be created using the marks we include, or Boolean combinations of marks. For each task, we show the marks and explanations of those marks in Table 5.

**Table 5:** Each MEG task with the temporal markers included in the dataset.

Artifact	*Onscreen Instruction*:	Go/No-Go	*Mark Explanation*
*blink*	“Please blink your eyes.”	*go*	Go stimulus
*eyemoveHoriz*	“Please move your eyes left and right.”	*nogo*	No-go stimulus
*eyemoveVert*	“Please move your eyes up and down.”	*response*	Button press response
*jawclench*	“Please clench your jaw.”	*response_hit*	Go stimulus with correct response*
*swallow*	“Please swallow.”	*response_miss*	Go stimulus with incorrect response*
*breath*	“Please take a deep breath.”	*response_correct_rejection*	No-go stimulus with correct response*
**Hariri Hammer**	***Mark Explanation***	*response_false_alarm*	No-go stimulus with incorrect response*
*encode_shape*	Shape target	**Sternberg**	***Mark Explanation***
*probe_shape*	Shape probe	*encode4*	4 character stimulus string
*encode_face*	Face target	*encode6*	6 character stimulus string
*encode_happy*	Face target – Happy emotion	*probe4*	Probe following 4 character stimulus
*encode_sad*	Face target – Sad emotion	*probe6*	Probe following 6 character stimulus
*encode_male*	Male face stimulus	*probe_in_set*	Probe character in preceding character string
*encode_female*	Female face stimulus	*probe_not_in_set*	Probe character not in preceding character string
*probe_face*	Face probe	*response_l*	Left button press
*probe_match_happy*	Face probe – Happy emotion match	*response_r*	Right button press
*probe_match_sad*	Face probe – Sad emotion match	*response_hit*	Probe (in set) with correct response
*response_hit*	Correct match	*response_miss*	Probe (in set) with incorrect rejection
*response_miss*	Incorrect match	*response_correct_rejection*	Probe (not in set) with correct response
*response_l*	Left response button	*response_false_alarm*	Probe (not in set) with incorrect response
*response_r*	Right response button	**Oddball**	***Mark Explanation***
**Airpuff**	***Mark Explanation***	*standard*	Standard (1 kHz tone)
*stim*	Airpuff stimulus trigger	*target*	Target (1.5 kHz tone)
*missingstim*	Missing stimulus trigger	*distractor*	Distractor (White noise)
**Movie**	***Mark Explanation***	*response*	Response
*trigger*	*Marks beginning of film presentation*.	*response_hit*	Correct response
		*response_miss*	Incorrect response

For the artifact scan the onscreen instruction is given; for all other tasks a brief explanation of the mark meaning appears in the table.

*For the go/no-go task, markers were used to encode trials with correct or incorrect responses. These marks temporally coincide with the presentation of the stimulus rather than the response for ease of analysis.

Most MEG data analysis software packages include in their standard pipeline co-registration of the MRI with the MEG using fiducial coils and head shape files. When sharing anonymized data, including MRIs missing anatomical landmarks, it is not immediately obvious how to circumvent this portion of the pipeline. In order to facilitate co-registration, the location of the anatomical landmarks are saved under the “AnatomicalLandmarkCoordinates” field within the MEG “coordsystem.json” sidecar file, which can be used to derive the appropriate transformations.

## Supplementary information

Supplementary Information

## Data Availability

MEG task paradigms, and scripts used for DICOM to BIDS format conversion and de-identification of structural MRI scans are available in the study git repository: https://github.com/nih-megcore/hv_protocol.
