# Supplementary material for: The NIMH intramural healthy volunteer dataset: A comprehensive MEG, MRI, and behavioral resource
Source: Sci Data. 2022 Aug 25;9:518. doi: 10.1038/s41597-022-01623-9 (PMC9403972; doi:10.1038/s41597-022-01623-9)
Supplement: Supplementary file 1 — Supplementary Information [file 41597_2022_1623_MOESM1_ESM.pdf]

Supplemental Table 1: Comparison of the NIMH Healthy Research Volunteer dataset demographics with that of the surrounding Montgomery County, Maryland, based on 2020 census data (https://data.census.gov).

	Montgomery County, MD	NIMH Dataset
Age		
Median Age	39.4	33
Minimum Age		18
Maximum Age		89
Sex		
% Female	51.6%	65.2%
Race		
White	76.3%	69.5%
Black/African American	13.4%	14.5%
American Indian/Alaska Native	1.3%	0.5%
Asian	5.9%	10.2%
Hawaiian/Pacific Islander	0.2%	0.0%
Mixed	2.8%	4.8%
Ethnicity		
Hispanic	18.5%	9.0%
Education		
Bachelors degree or above	59.2%	84.9%
Income		
<10k	3.40%	6.20%
10-15k	1.80%	2.00%
15-25k	4.00%	2.80%
25-35k	4.80%	11.90%
35-50k	6.70%	7.80%
50-75k	12.50%	17.90%
75-100k	11.60%	13.90%
100-150k	19.20%	16.30%
150-200k	12.70%	9.70%
>200k	23.50%	11.60%
Employment		
In civilian labor force	70.5%	
full-time		62.0%
part-time		11.1%
student		11.4%
Work on NIH campus		11.4%

Supplemental Table 2: Name of each MRI scan and the number of participants completing each for data shared in the sourcedirectory. Note that parameters may not be uniform across all images; relevant parameters can be exctracted from the included .json files.

Modality	Series Description	Image Type	Count
T1w	acq-MPRAGE_rec-SCIC	only SCIC available	2
T1w HighResHipp	acq-HighResHippo_T1w		2
2D FLAIR	acq-2d_rec-SCIC_FLAIR	only SCIC available	12
3D FLAIR	acq-Sagittal_rec-SCIC or acq-VariableTE_rec-SCIC	only SCIC available	22
T2*	rec-SCIC_T2starw	only SCIC available	8
ASL	asl	eyes open	12
DTI	dir-unflipped_dwi	24 directions (reverse)	15
	dir-flipped_dwi	48 directions (primary)	20
rsfMRI	dir-forward_bold	Forward	11
	dir-reverse_bold	Reverse	1

Supplemental Table 2: Details regarding the timing of each MEG task paradigm. Total durations are approximate due to variable jittering in the timing for each trial.

	Trial Categories	Number of Blocks	Trials per block	Total Trials	Trial Components	Stimulus Duration	Post-stim fixation/ISI	Total Duration (approximate)
Rest								6 min
Naturalistic Viewing	Movie "Groei/Growth"							8.5 min
Artifact	Prompt			6		2s	2s	24 s
Oddball	Standard			210		50ms	950ms + jitter	5.67 min
	Target			45		50ms	950ms + jitter	
	Distractor			45		50ms	950ms + jitter	
Go/No-go	Go			200		200ms	967ms	5.8 min
	No-Go			100		200ms	967ms	
Airpuff	Stim			425		50ms	450ms	4 min
	Missing Stim			75		50ms	450ms	
Hariri Hammer	Faces	3	30	90	Target	1000ms	500ms	10.3 min
					Probe	1000ms	1500ms + jitter	
	Shapes	4	15	60	Target	1000ms	500ms	
					Probe	1000ms	1500ms + jitter	
Sternberg	4 Character String	4	10	40	Target	2000ms	3000ms	11 min
					Probe	1000ms	1500ms	
	6 Character String	4	10	40	Target	2000ms	3000ms	
					Probe	1000ms	1500ms	
